# Acute Liver Failure Secondary to Niacin Toxicity

**DOI:** 10.1155/2014/692530

**Published:** 2014-02-12

**Authors:** Marc A. Ellsworth, Katelyn R. Anderson, David J. Hall, Deborah K. Freese, Robin M. Lloyd

**Affiliations:** ^1^Department of Pediatric and Adolescent Medicine, Mayo Clinic, Rochester, MN 55905, USA; ^2^Division of Neonatal Medicine, Mayo Clinic, Rochester, MN 55905, USA; ^3^Division of Pediatric Gastroenterology, Mayo Clinic, Rochester, MN 55905, USA; ^4^General Pediatrics and Adolescent Medicine, Mayo Clinic, Rochester, MN 55905, USA

## Abstract

A 17-year-old male was transferred to the pediatric intensive care unit for evaluation of acute liver failure. He was recently released from an alcohol treatment center with acute onset of chest pain. Cardiac workup was negative but he was found to have abnormal coagulation studies and elevated liver transaminases. Other evaluations included a normal toxicology screen and negative acetaminophen level. Autoimmune and infectious workups were normal providing no identifiable cause of his acute liver failure. He initially denied any ingestions or illicit drug use but on further query he admitted taking niacin in an attempt to obscure the results of an upcoming drug test. Niacin has been touted on the Internet as an aid to help pass urine drug tests though there is no evidence to support this practice. Niacin toxicity has been associated with serious multisystem organ failure and fulminant hepatic failure requiring liver transplantation. Pediatric providers should be aware of the risks associated with niacin toxicity and other experimental medical therapies that may be described on the Internet or other nonreputable sources.

## 1. Introduction

The Internet as a source of medical information and advice impacts the care that pediatricians provide for their patients [1, 2]. There is information available to teenagers on different supplements that can be consumed to mask illicit drug testing. The consequences are not readily considered by adolescents because they see them as easy to obtain over-the-counter medications; however, some of these supplements can be toxic or even lethal [3]. We present the case of an adolescent male who presented to our pediatric intensive care unit (PICU) with acute liver failure (ALF) secondary to niacin toxicity that was ingested as an effort to mask an upcoming drug screen.

## 2. Case

A-17-year old male was transferred from an outside medical facility to the PICU for evaluation of ALF. He was recently released from an alcohol treatment center and presented to the outside hospital with acute onset of chest pain. Cardiac workup was negative but he was found to have significant laboratory abnormalities including abnormal coagulation studies with an international normalized ratio of 4.8 (normal 0.8–1.2), prothrombin time of 32.3 seconds (normal 9.5–13.8), and activated partial thromboplastin time of 47 seconds (normal 28–38 seconds). Liver transaminases were elevated with an aspartate transaminase of 295 U/L (normal 8–48 U/L), alanine transaminase of 284 U/L (normal 7–55 U/L), and total bilirubin of 8.4 mg/dL (normal 0.1–1.0 mg/dL) with a direct bilirubin of 1.9 mg/dL (normal 0.0–0.3 mg/dL). Other evaluations included a normal X-ray computed tomography and ultrasound of the abdomen, normal toxicology screen for drugs of abuse, and negative acetaminophen level. Complete blood count, electrolytes, and renal function were normal. Albumin, ammonia, lipase, amylase, gamma glutamyl transpeptidase, hepatitis viral screen, and ceruloplasmin were normal. He had no constitutional signs of any acute illness and initially denied any ingestions or illicit drug use.

Transport was without event and he received two units of fresh frozen plasma as well as vitamin K for his coagulopathy en route. During his hospital stay, he showed rapid improvement in his acute liver injury and was able to be transferred to the general pediatric service within two days. He demonstrated some fluctuations but with overall improvement in his liver function. Additional testing for autoimmune markers, infectious etiologies, and alpha-1 antitrypsin was negative. On further query, he admitted ingesting an unspecified amount of over-the-counter niacin in an attempt to obscure the results of an upcoming drug test. He reported using the Internet as his source of information on the use of niacin as a possible way to mask his recent use of marijuana on the drug screen. His ALF was attributed to this use of niacin and no further workup was undertaken. He continued to improve rapidly and within two weeks his liver transaminases and coagulation studies returned to normal.

## 3. Discussion

Our case is a unique description of an adolescent presenting to our hospital with ALF secondary to niacin induced toxicity. This clinical case offers many valuable insights and learning opportunities for those providers caring for the adolescent population. Among these are a review of the broad differential diagnosis for pediatric ALF, an understanding of the role that the Internet plays in guiding medical related practices of our patient population, and a reminder of thorough and repeated history taking in this unique population.

ALF is defined as biochemical evidence of acute liver injury and hepatic-based coagulopathy in patients with no known evidence of chronic liver disease. ALF can range from fulminant hepatic failure requiring significant medical management and eventual transplant to milder cases that only require initial supportive management with eventual resolution and recovery. What is specifically difficult for hospital practitioners in the setting of ALF is determining the etiology of the liver injury, a task which is important in helping determine treatment and prognosis in such patients.

In 1999 the Pediatric Acute Liver Failure Study Group was formed to prospectively study children worldwide in an attempt to better study the pathogenesis and outcomes in pediatric ALF. Of those diagnosed with ALF (inclusion criteria: no evidence of chronic liver disease; biochemical evidence of acute liver injury; hepatic-based coagulopathy plus/minus hepatic encephalopathy) only about 50% had an identifiable cause [4]. Of those who had etiologies identified, most causes could be attributed to one of four categories: metabolic, infectious, autoimmune, and toxic/drugs.

Metabolic disorders account for approximately 10% of pediatric ALF and can either present initially with liver failure or with other systemic complications. Representative disorders include galactosemia, hereditary tyrosinemia type 1, hereditary fructose intolerance, fatty acid oxidation disorders, mitochondrial disorders, and rarely inborn errors of bile acid synthesis. Although often tested for and part of the differential diagnosis, infectious etiologies are very rarely the cause of ALF in the pediatric population with Epstein-Barr virus being the most commonly identified culprit. Autoimmune causes, such as sclerosing cholangitis, are difficult to diagnose but often prompt extensive serologic evaluations when the cause of ALF is undetermined.

The last, and significantly most common, category of pediatric ALF is toxic/drugs. Approximately 20% of pediatric cases of ALF are drug induced with the vast majority of these cases being a result of ingestion of acetaminophen. However, in our case we describe significant liver damage caused by ingestion of an unknown amount of over-the-counter niacin. Niacin, vitamin B3, is a readily available supplement typically utilized for treating dyslipidemia and niacin deficiency. Niacin toxicity has been associated with serious multisystem organ damage and fulminant hepatic failure requiring liver transplantation [5, 6]. The slow release preparation is associated with the highest risk of liver toxicity [7].

**Figure 1:**
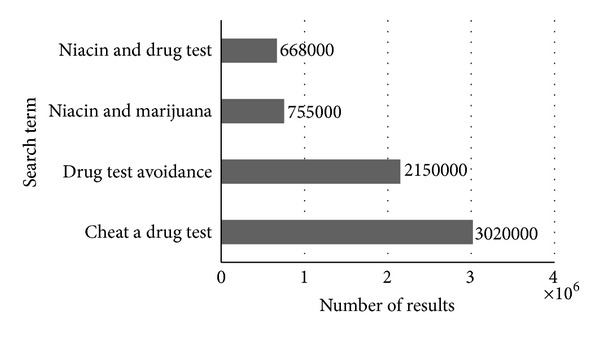
Results of a Google search regarding topics related to niacin and drug tests.

The availability of niacin as an over-the-counter supplement makes it readily available to our patient population with no oversight of its use by a physician, parent, or guardian. In addition, there have been concerns raised over the use of supplemental preparations of niacin as such products have less stringent US Food and Drug Administration regulations [3]. The use of niacin as a drug test evasion technique is highly touted on the Internet with its possible efficacy readily championed on a myriad of electronic resources. A simple Google search using keywords related to this topic returned over six million search results (Figure 1) [8]. Interestingly, our patient's urine drug screen was negative despite acknowledgement of recent marijuana use.

Our case is just one example of how the use of the Internet for dissemination of medical information can be harmful to our patient population. One study evaluated multiple Internet sources that guide parents on how to treat the common cough in children, with over half of the sites recommending treatments discouraged by the American Academy of Pediatrics [1]. Burgos et al. determined that approximately 25% of all edits that were made to specific medically related Wikipedia pages were classified as potentially controversial [2]. In addition, a systemic review of studies evaluating the quality of consumer health information on the World Wide Web found that 70% of the studies concluded that quality is a problem on the web [9].

Regardless of the mounting evidence suggesting the unreliability of Internet-obtained health information, it remains a significant component of patient guided healthcare. One study estimates that more than half of patients use the Internet for health information with nearly 60% not discussing this information with their doctors [10]. Even though much of this research has been conducted in the adult population, it is safe to assume that, due to the ubiquitous access to the Internet among our adolescent population, similar trends would occur in this unique population. It is imperative for pediatricians to be aware of this tool and how patients are accessing it for medical knowledge. Constant updates of the trends and recent fads in Internet-based medical information can be very helpful in educating physicians on what information their patients are exposed to, information that is often not shared with the physician.

The last insightful point that our case illustrates is a reminder of the need for thorough and often repeated history taking in our adolescent patients. Our specific patient was interviewed multiple times, by multiple providers (attending, senior and junior residents and a medical student) without any disclosure of his ingestion of niacin. However, on further history taking by the senior resident, in the absence of family and friends, the patient openly volunteered information regarding his recent use of niacin and purpose of its use. Only then could a presumptive cause of his ALF be determined with discontinuation of the extensive diagnostic evaluation.

## 4. Conclusion

The differential diagnosis for ALF in the pediatric population is broad and often an underlying etiology is not identified. However, as seen in our case, extensive history taking and an understanding of Internet trends may aid in determining the causes of disease presentation in the adolescent population. Our patient developed ALF secondary to the use of niacin as a way to help mask the results of an upcoming urine drug screen. Awareness of this and similar practices is vital to providers giving medical care to this population.

## Abbreviations

ALF: Acute liver failure
PICU: Pediatric intensive care unit.
